# Cow’s Milk as a Vehicle of Disease

**Published:** 1884-12

**Authors:** 


### COW'S MILK AS A VEHICLE OF DISEASE.

The possibility of the transmission of infectious and epidemic diseases through the agency of cow's milk has become a well recognized fact. Since Dr. Ballard published his report of an epidemic of typhoid fever at Islington, in 1870, attention has been directed to this source of dissemination of disease, and the result has been a record of at least one hundred epidemics alleged, upon reliable grounds, to have been traceable to milk which had in some way or another become specifically contaminated.

It seems to be an established fact that scarlet fever has been communicated in this manner, and there is reason for supposing that diphtheria has also been thus disseminated, although the evidence on this latter point is not so thoroughly conclusive. Other infectious diseases are believed to have been occasionally propagated through milk, but more proof is needed to reduce the opinion to one of scientific accuracy. However, as we are in possession of certain well-known facts in regard to this mode of disseminating typhoid fever and scarlet fever, and knowing what we do of the nature of infectious diseases, and with our knowledge of the property in milk of readily absorbing volatile matters in the atmosphere, and of the circumstances attending the collection, treatment and handling of milk before it reaches the consumer, it is not hazardous to venture the opinion that all infections may be transmitted by milk, and that this possible source of danger to health should be guarded against accordingly.

It is known that milk containing a fungus—*Odium lactis* or *penicillium*—may give rise to irritation of the stomach, or even gastritis. Milk from an inflamed udder will cause inflammation of the mucous membrane of the mouth and aphthæ on the lips and gums. The so-called milk sickness, at one time prevalent in the Western States, is supposed to have been caused by the milk of cows which fed on the *Rhus Toxicodendron.*

Very positive evidence has been adduced to show that the milk of cows affected with the foot-and-mouth disease will give rise to a somewhat similar affection in the human subject. It is not so clear how milk becomes the means of conveying the poison of enteric fever, scarlet fever, and possibly some other infectious diseases. In the case of typhoid fever communicated in this way, the majority of epidemics have been regarded as due to specifically contaminated water which had been added to the milk. In other instances of typhoid fever, and in the case of scarlet fever, and perhaps diphtheria, a common explanation is that the infectious material has been absorbed by the milk. It has also been suggested that the milk thus infected may act, while warm, as a cultivation-fluid for the zymotic germs. Other explanations have been proposed, but they do not materially modify the general precautions which, in the present state of our knowledge, are deemed most efficacious in preventing this mode of transmission of disease.

Dr. Thursfield, an English medical officer of health, who has investigated the subject of milk epidemics very carefully, proposes certain precautions which he considers effectual in preventing these outbreaks of disease. The responsibility is divided between the consumer and the sanitary authorities. He urges upon the consumer the precaution of boiling all milk. There is a prejudice against this practice, but it ought to give way if it be true that "to boil milk may, for practical purposes, be said to confer immunity from infection conveyed by it."

The milk-shop of the retailer and the dairy of the wholesale purveyor should be placed under the strict control of the sanitary authorities, which should be clothed with power to make proper regulations and to enforce them by the aid of efficient inspection. The organization of such a service would at first be arduous, but so soon as its requirements are made known and intelligently comprehended, a willing co-operation might be expected in most cases. There is a prevailing ignorance of the facts above stated, which is damaging to the best interests of the public health, and ought to be removed. In no way can this be better accomplished than by the organization of an authoritative service regulating the purveying and sale of this important article of food.—*Med. News, Nov.* 29.

